# Adjuvantic cytokine IL-33 improves the protective immunity of cocktailed DNA vaccine of ROP5 and ROP18 against *toxoplasma gondii* infection in mice

**DOI:** 10.1051/parasite/2020021

**Published:** 2020-04-21

**Authors:** Yu-Chao Zhu, Yong He, Jian-Fa Liu, Jia Chen

**Affiliations:** 1 Ningbo University School of Medicine Ningbo Zhejiang Province PR China; 2 Department of Otorhinolaryngology, The Affiliated Hospital of Ningbo University Medical School Ningbo Zhejiang Province PR China

**Keywords:** *Toxoplasma gondii*, Toxoplasmosis, IL-33, Rhoptry protein 5 (ROP5), Rhoptry protein 18 (ROP18), DNA vaccine, Protective immunity

## Abstract

*Toxoplasma gondii* is a threat for immunocompromized individuals, and no treatment is available for enhancing immunity against infection. Molecular adjuvants may improve the efficacy of DNA vaccine-induced T cell immunity. Here, we report that cocktailed DNA immunization with ROP5 and ROP18 boosted immune responses induced by a single DNA immunization with ROP5 or ROP18, but also that co-administration of molecular adjuvant IL-33 enhanced immune efficacy induced by this cocktailed DNA vaccination. These improved immune responses were characterized by higher *Toxoplasma*-specific IgG2a titers, Th1 responses associated with the production of IFN-γ, IL-2, IL-12, as well as cell-mediated activity with higher frequencies of CD8+ and CD4+ T cells. More importantly, this enhanced immunity has the ability to confer remarkable protection against a high dose lethal challenge of the *T. gondii* RH strain and thus against chronic infection with the *T. gondii* PRU strain. These data show that IL-33 is a promising immunoadjuvant to facilitate humoral as well as cellular immunity in a vaccine setting against *T. gondii*, and suggest that it should be evaluated in strategies against other apicomplexan parasites.

## Abstract (fr)

*Toxoplasma gondii* est une menace pour les individus immunodéprimés et aucun traitement n’est disponible pour renforcer l’immunité contre l’infection. Les adjuvants moléculaires peuvent améliorer l’efficacité de l’immunité des cellules T induite par un vaccin à ADN. Ici, nous rapportons que l’immunisation par le cocktail d’ADN de ROP5 et ROP18 a stimulé les réponses immunitaires induites par une seule immunisation par l’ADN de ROP5 ou ROP18, mais aussi que la co-administration de l’adjuvant moléculaire IL-33 a amélioré l’efficacité immunitaire induite par cette vaccination par cocktail d’ADN. Ces réponses immunitaires améliorées ont été caractérisées par des titres d’IgG2a spécifiques à *Toxoplasma* plus élevés, des réponses Th1 associées à la production d’IFN-γ, IL-2, IL-12 ainsi qu’une activité à médiation cellulaire où les fréquences des cellules T CD8+ et CD4+ étaient plus élevées. Plus important encore, cette immunité renforcée a la capacité de conférer une protection remarquable contre une provocation létale par haute dose de la souche RH de *T. gondii* et donc contre une infection chronique par la souche PRU de *T. gondii*. Ces données montrent qu’IL-33 est un immunoadjuvant prometteur pour faciliter l’immunité humorale et cellulaire dans un contexte de vaccination contre *T. gondii* et suggèrent qu’il devrait être évalué dans des stratégies contre d’autres parasites apicomplexes.

## Introduction

*Toxoplasma gondii* is an obligate intracellular parasite that can infect most warm-blooded animals, leading to zoonotic infections [11, 14, 23]. By invading and replicating in nucleated cell in immunocompromized individuals and unborn fetuses, *T. gondii* can cause severe disseminated disease in adults and congenital defects in newborns, respectively [22, 33, 34]. Toxoplasmosis also may cause abortion and neonatal loss in livestock, resulting in considerable economic losses, especially in pigs and sheep [28]. Due to the fact that chemical treatments are insufficient to eliminate tissue cysts, as well as residual drug in food and drug-resistant parasites, the development of a vaccine is a public health priority as an alternative strategy against *T. gondii* infection.

Current vaccine designs have focused on the identification of candidates against toxoplasmosis in mice models, including a variety of virulence factors of *T. gondii*, consisting of surface antigens (SAGs), micronemal (MIC) proteins, dense-granule antigens (GRAs), and rhoptry proteins (ROPs) [37, 39]. Among these well-defined antigens, ROP18, ROP16, ROP5, and ROP17 have been identified as major virulence loci mediating *T. gondii* virulence in mice [8, 10]. Recent studies have shown that both the ROP5 and ROP18 alleles are key murine virulence factors across global *T. gondii* strains [26]. Moreover, cocktailed DNA vaccination has yielded more promising protective efficacy than that of single-gene DNA immunization [3, 39]. As a result, these encouraging findings have led us to assume that DNA vaccination with ROP5 and ROP18 may evoke robust protective responses against toxoplasmosis in mice, which may be comparable to host responses induced by DNA immunization with GRA15 and ROP5 [3].

Since adjuvants are known to shape the quantity and quality of immune responses, they are commonly used as critical components in most clinical vaccines, and are thus used to drive and specifically direct the desired responses [25]. Currently, DNA-based vaccines in conjunction with cytokine adjuvants have been developed, and this strategy has also been found to be particularly promising for boosting the adaptive immune response against infectious disease or antitumor cell-mediated immune responses [6, 27]. Our previous attempts to incorporate adjuvant cytokines have been shown to help improve the potency of DNA-based vaccines, such as IL-18, IL-21/IL-15, and IL-7/IL-15 [4, 17, 36]. Therefore, molecular adjuvants including cytokines, are actively being recognized as an ideal way to improve the efficacy of vaccines. As a member of the IL-1 cytokine family, IL-33 has emerged as a pro-inflammatory cytokine and has recently been reported to drive protective antiviral CD8+ T cell responses [2, 30]. Moreover, IL-33 can act as a novel immunoadjuvant to augment vaccine-induced protective antiviral CD8+ T cell responses and thus to improve T cell responses induced by an anti-tumor DNA vaccine [29, 31].

Therefore, the objectives of this study were to determine whether DNA vaccination with pVAX-ROP5 and/or pVAX-ROP18 could prime the protective immunity against infection with two different *T. gondii* strains in mice. Also, we constructed eukaryotic expression plasmids pVAX-IL-33, to assess the immune-enhancing effect of cytokine adjuvants by co-delivery with pVAX-ROP5 and pVAX-ROP18.

## Materials and methods

### Animals and parasites

Six- to eight-week-old specific pathogen-free female Kunming mice were purchased from Zhejiang Laboratory Animal Center, Hangzhou, China. All animals were maintained in strict accordance with the Good Animal Practice requirements of the Animal Ethics Procedures and Guidelines of the People’s Republic of China. Animal experiments were approved by the ethics committee of Ningbo University (permission: SYXK(ZHE)2013-0191).

Tachyzoites of the *T. gondii* RH strain (type I) were used for this study, and were maintained in our laboratory and prepared from human foreskin fibroblast (HFF) cells. HFF cells were cultured with Dulbecco’s modified Eagle’s medium (DMEM) supplemented with 2% fetal bovine serum (FBS) (Gibco, Carlsbad, CA, USA). The obtained tachyzoites were used for total RNA extraction (RNApre Pure Tissue Kit, Sangon Biotech, China) and the preparation of *Toxoplasma* lysate antigen (TLA), as described in our previous studies [17]. The tissue cysts of the low-virulence PRU strain were propagated and harvested, as described in our previous studies [4], and were used for mice challenge.

### Construction of the eukaryotic expression plasmids

Total RNA was isolated from spleens of Kunming mice, and was prepared as described previously, by using TRIzol reagent (Invitrogen), according to the manufacturer’s instructions. The complete open-reading frame of IL-33 was obtained by RT-PCR amplification from total RNA using specific primers F1 (forward primer: 5′ – GG**GGTACC** ATGACGTCGCAGCTCGCTACT – 3′, reverse primer: 5′ – GC**TCTAGA** CACTGAACTGATAGGCGCAG – 3′), in which *Kpn* I and *Xba* I restriction sites were introduced (bold).

The obtained PCR product was inserted into the pMD-18 T Vector (TaKaRa, China), generating pMD-IL-33. The IL-33 fragments were cleaved by *Kpn* I and *Xba* I from pMD-IL-33 and then subcloned into pVAX I (Invitrogen), which was cleaved by *Kpn* I and *Xba* I, by T4 DNA ligase to generate plasmid pVAX-IL-33. The recombinant plasmids were identified by PCR, double restriction enzyme digestion, and sequencing. The positive plasmids were purified from transformed *Escherichia coli* DH5α cells by anion exchange chromatography (EndoFree plasmid giga kit, Qiagen Sciences, MD, USA), according to the manufacturer’s instructions. The concentrations of plasmids were determined by spectrophotometer at OD260 and OD280, and then dissolved in sterile phosphate-buffered saline (PBS) with a final concentration of 1 mg/mL and stored at –20 °C until use.

### Expression of recombinant plasmid *in vitro*

In order to confirm the expression of pVAX-IL-33 *in vitro*, HEK 293-T cells were transfected with recombinant plasmids pVAX-IL-33 using LipofectamineTM 2000 reagent (Invitrogen), according to the manufacturer’s instructions. As the negative control, HEK 293-T cells were transfected with empty pVAX I. At 48 h after transfection, the supernatants of the cells transfected with pVAX-IL-33 were tested using ELISA kits according to the manufacturer’s instructions (Mouse IL-33 ELISA Kit, abcam), as previously described [4, 17].

### Immunization and challenge

A total of eight groups of Kunming mice were used for this experiment (Table 1). Among these groups, five (*n* = 25 in each group) were immunized with 100 μg of plasmid DNA dissolved in 100 μL sterile PBS, and there were three control groups (blank, PBS, and empty pVAX I vector control). For DNA immunization, mice were inoculated intramuscularly at the bilateral quadriceps with the same dose three times, at a two-week interval.

**Table 1:** Summary of treatments performed in study mice.

Group	Treatments (of 100 μL PBS)	Total sample size	Route of administration	Sample size in HI^a^	Sample size in CMI^b^	Sample size in challenges
I	Control	25	–	3	9	10^c^, 6^d^
II	100 μL PBS	25	Thigh muscle	3	9	10^c^, 6^d^
III	100 μg pVAX1	25	Thigh muscle	3	9	10^c^, 6^d^
IV	100 μg pVAX-IL33	25	Thigh muscle	3	9	10^c^, 6^d^
V	100 μg pVAX-ROP5	25	Thigh muscle	3	9	10^c^, 6^d^
VI	100 μg pVAX-ROP18	25	Thigh muscle	3	9	10^c^, 6^d^
VII	50 μg pVAX-ROP5 + 50 μg pVAX-ROP18	25	Thigh muscle	3	9	10^c^, 6^d^
VIII	33 μg pVAX-IL33 + 33 μg pVAX-ROP5 + 33 μg pVAX-ROP18	22	Thigh muscle	3	9	10^c^, 6^d^

a To assess humoral immunity (HI), sera were collected from the tail vein prior to immunization from three mice per group.

b To assess cell-mediated immunity (CMI), through the lymphocyte proliferation assay (*n* = 3 mice), cytokine measurements (*n* = 3 mice), and flow cytometric analysis (*n* = 3 mice), spleens were aseptically removed from nine mice per group two weeks after the last immunization.

c Two weeks after the final inoculation, the mice in all groups were challenged intraperitoneally with 1 × 10^3^ tachyzoites of *T. gondii* RH strain.

d Two weeks after the final inoculation, the mice in all groups were challenged intragastrically with 20 cysts of *T. gondii* PRU strain.

Two weeks after the last immunization, 10 mice per group were challenged intraperitoneally with 1 × 10^3^ tachyzoites of the highly virulent *T. gondii* RH strain, as the acute challenge infection, and their survival was recorded daily until all mice had died. As the chronic challenge infection, another six mice in each group were inoculated orally with 6 PRU tissue cysts, and the mean number of cysts per brain was determined, as described in our previous studies [3, 40]. The parasite reduction rate of brain cysts is relative to that of the blank control.

Two weeks after the last immunization, a total of nine mice per group were sacrificed and splenocytes were aseptically harvested for flow cytometric analysis (*n* = 3 mice), measurement of spleen cell proliferation (*n* = 3 mice), and cytokine (*n* = 3 mice).

### *Toxoplasma gondii*-specific antibody response

Blood samples were collected from the mouse tail vein prior to each immunization and challenge (at weeks 0, 2, 4, and 6), and serum samples were separated after clot retraction and stored at –20 °C until analysis. Pre-immune (at weeks 0) serum samples were used as negative controls. The levels of anti-*T. gondii* IgG, IgG1 and IgG2a in serum samples were detected using an SBA Clonotyping System-HRP Kit (Southern Biotech Co., Ltd., Birmingham, AL, USA), according to the manufacturer’s instructions and as previously described [3]. The absorbance was measured at 405 nm using ELISA reader (Bio-TekEL × 800, USA). All samples were run in triplicate.

### Spleen cell proliferation to *T. gondii* soluble antigen

Two weeks after the last immunization, spleens from three mice in each group were aseptically removed. The splenocytes were harvested by pushing the spleens through a nylon net (200 meshes), and lysed by erythrocyte lysis buffer solution (Sangon, China). The purified splenocytes were adjusted in density to 2 × 10^5^ cell/mL, and were re-suspended in DMEM medium supplemented with 2% fetal calf serum (FCS). Then, the splenocytes were plated onto 96-cell plates and cultured with TLA (10 μg/mL), concanavalin A (Con A; 5 μg/mL) or medium alone used as positive and negative controls, respectively. After incubation for 44 h at 37 °C in a 5% CO_2_, 10 μL of CCK-8 reagent (Enhanced Cell Counting Kit-8, Beyotime, China) were added to each well and the splenocytes were incubated for a further 4 h. Proliferative activity was evaluated by measuring absorbance at 570 nm. The stimulation index (SI) for each group = (OD570TLA/OD570pVAX): (OD570ConA/OD570pVAX). All experimental and control samples were run in triplicate.

### Characterization of spleen lymphocytes by flow cytometry

Lymphocytes were isolated from spleen, as described above. According to the method described in our previous studies [3, 40], surface staining was performed by incubation with surface markers, including phycoerythrin-(PE)-labeled anti-mouse CD3, allophycocyanin (APC)-labeled anti-mouse CD4, and fluorescein isothiocyanate (FITC)-labeled anti-mouse CD8 (eBioscience). The cell suspension was then fixed with FACScan buffer (PBS containing 1% BSA and 0.1% sodium azide) and 2% paraformaldehyde. All data were acquired through a FACScan flow cytometer (BD Biosciences, USA). The analysis was performed with the data from three independent experiments.

### Cytokine assay

Splenocytes harvested from each group were co-cultured with TLA (10 g/mL) and medium alone (negative control) in flat-bottom 96-well microtiter plates. Culture supernatants were harvested and assayed for IL-2 and IL-4 at 24 h, IL-10 at 72 h and IL-12(p40), IFN-γ and IL-12 (p70) at 96 h using commercial ELISA kits, according to the manufacturer’s instructions (BioLegend, USA) and previous studies [3, 40]. The analysis was performed with the data from three independent experiments.

### Statistical analysis

All statistical analyses were processed by SPSS18.0 Data Editor (SPSS, Inc., Chicago, IL, USA). The differences in antibody responses and lymphoproliferation assays, cytokine production, percentages of CD4+ and CD8+ T cells, and brain cyst loading were compared between groups by one-way ANOVA. The differences were considered statistically significant if *p* < 0.05.

## Results

### Identification of IL-33 gene expression *in vitro*

By using capture ELISA kits, the expression of pVAX-IL-33 was confirmed *in vitro*, in accordance with the measurement range 31.2 pg/mL – 2000 pg/mL for IL-33. High levels of IL-33 (347 pg/mL) were detected in the supernatant of HEK 293-T cells transfected with pVAX-IL-33, while no detected levels of IL-33 were observed in cells transfected with the empty pVAX I.

### Antibody detection

**Figure 1:**
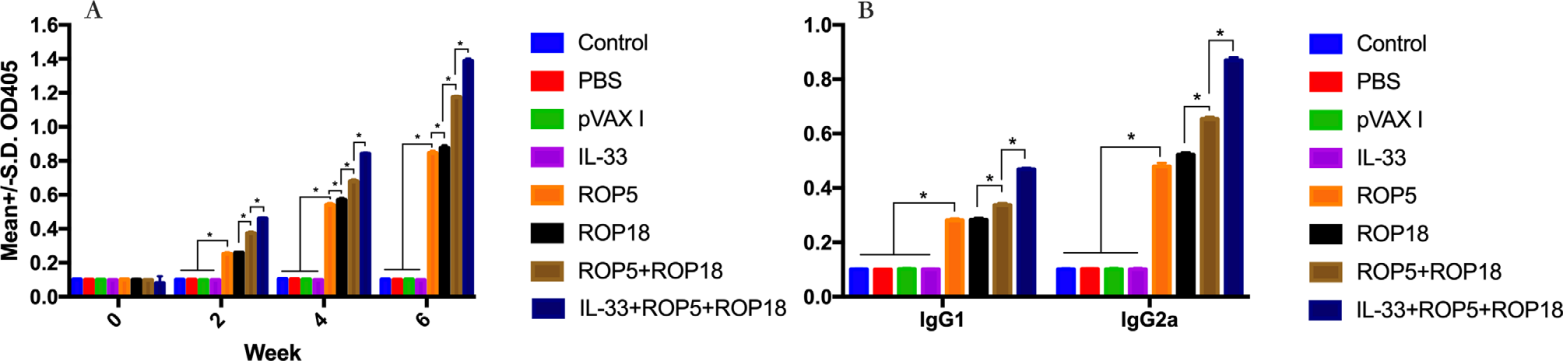
Detection of IgG, IgG1, and IgG2a antibodies in the sera of Kunming mice. (A) Determination of IgG antibodies induced by DNA immunization at weeks 0, 2, 4, 6. (B) Determination of IgG1 and IgG2a in the sera of mice two weeks after the last immunization. Results are expressed as means ± SD (*n* = 3) with respect to absorbance at OD405 and statistical differences (*p* < 0.05) are indicated by (*). The bars represent the levels of IgG, IgG1, and IgG2a in the serum of mice.

To investigate humoral immune response induced by all immunized mice, serum samples were obtained prior to each immunization and challenge (at weeks 0, 2, 4, and 6), and the titers of IgG and subclasses IgG (IgG1 and IgG2a) in the experimental groups and the three control groups were detected by standard ELISA. As shown in Figure 1A, statistically significantly higher levels of IgG were found in the sera of all experimental groups, and specific antibody levels did significantly increase with continuous immunization, while no increase of antibody titers occurred among the three control groups. It was also shown that the highest antibody levels were in the group that received pVAX-IL-33 + pVAX-ROP5 + pVAX-ROP18, intermediate antibody levels in the group that received pVAX-ROP5 + pVAX-ROP18, and the lowest antibody levels in the sera of mice immunized with pVAX-ROP5 or pVAX-ROP18 alone. However, no significant difference was observed between the pVAX-ROP5 and pVAX-ROP18 groups (*p* > 0.05).

As shown in Figure 1B, the levels of IgG1 and IgG2a, and thus the ratio of IgG2a – IgG1 were significantly increased in the immunized mice, in contrast to the three control groups (blank control, PBS and pVAX I) (*p* < 0.05). These results indicated that a predominant Th1 type immune response was elicited successfully. Meanwhile, the ratios of IgG2a/IgG1 were higher in mice immunized with pVAX-ROP5 + pVAX-ROP18 in comparison with those with single DNA immunization with pVAX-ROP5 or pVAX-ROP18. Co-administration of pVAX-IL-33 with pVAX-ROP5 + pVAX-ROP18 induced the highest IgG2a/IgG1 ratio. However, no significant difference in the IgG2a/IgG1 ratio was observed between the pVAX-ROP5 and pVAX-ROP18 groups (*p* > 0.05).

### Splenocyte proliferation

To analyze the proliferation of splenocytes, splenocytes from immunized mice and non-immunized mice were harvested two weeks after the last immunization, and the assay was carried out by stimulation of splenocytes with TLA or ConA. As shown in Table 2, the highest lymphocyte proliferation stimulation index (SI) was found in the group of mice immunized with pVAX-ROP5 + pVAX-ROP18 in comparison with those from mice immunized pVAX-ROP5 or pVAX-ROP18 alone (*p* < 0.05). Co-injection with pVAX-IL-33 and pVAX-ROP5 + pVAX-ROP18 enhanced the SI of pVAX-ROP5 + pVAX-ROP18 significantly. However, no significant difference in SI was observed between the pVAX-ROP5 and pVAX-ROP18 groups (*p* > 0.05).

**Table 2:** Proliferative response of lymphocytes of immunized Kunming mice after stimulation by *Toxoplasma gondii* lysate antigen.

Group (*n* = 3)	Proliferation (SI)
pVAX-IL-33 + pVAX-ROP5 + pVAX-ROP18	5.58 ± 0.07^A^
pVAX-ROP5 + pVAX-ROP18	4.37 ± 0.05^B^
pVAX-ROP18	3.39 ± 0.05^C^
pVAX-ROP5	3.11 ± 0.07^C^
pVAX-IL-33	1.32 ± 0.04^D^
pVAX I	1.02 ± 0.03^D^
PBS	1.03 ± 0.03^D^
Control	1.02 ± 0.03^D^

*Note*: The same letter means that the data are not statistically significantly different in the column, and different letters means that the data are statistically significantly different in the column.

### Flow cytometry analysis of lymphocytes subpopulations

**Figure 2:**
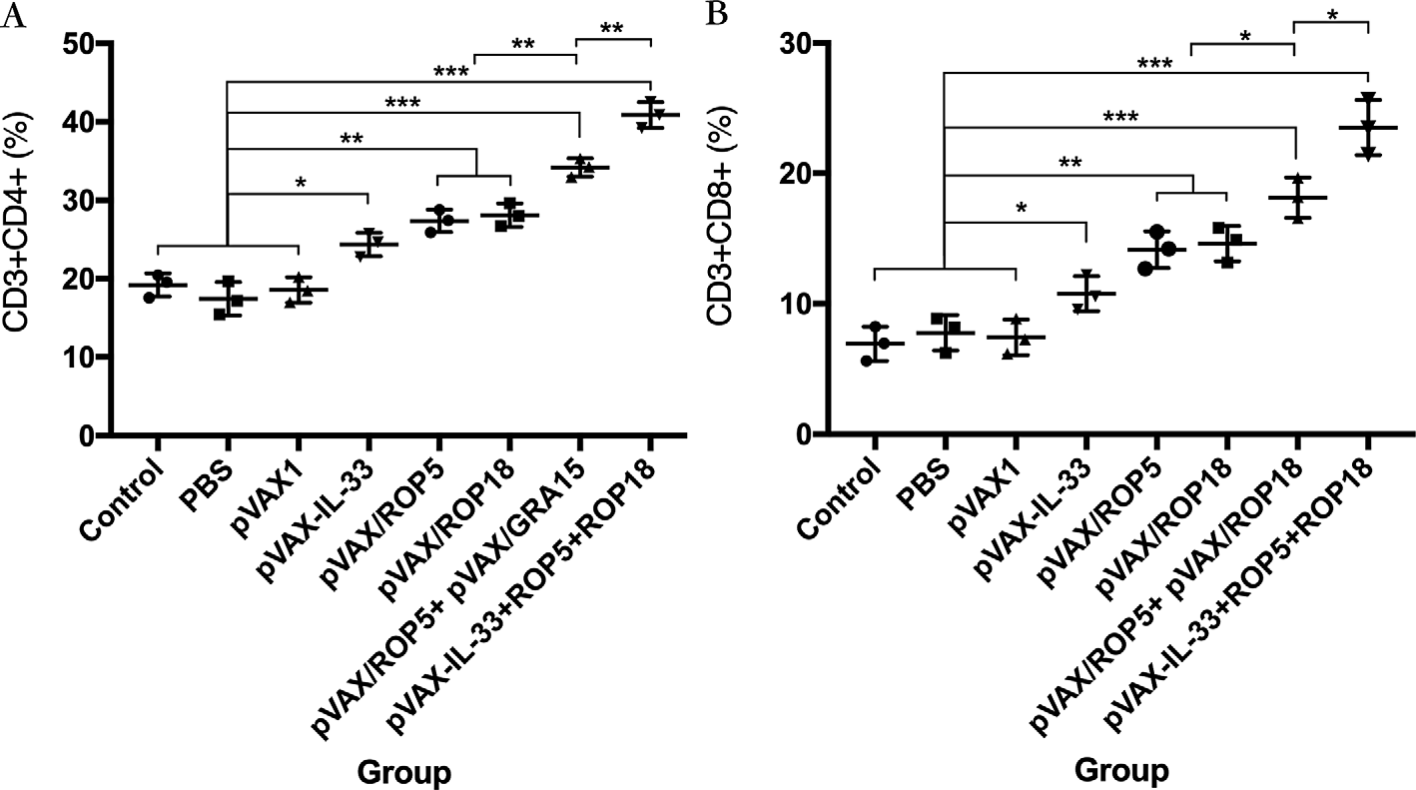
DNA immunization augmented the frequency of antigen-specific T cells in mice. (A) Total numbers of CD3+ CD4+ CD8− T lymphocytes per spleen. (B) Total numbers of CD3+ CD8+ CD4− T lymphocytes per spleen. Data are means ± SD (representative of three experiments). *p < 0.05, **p < 0.01, and ***p < 0.001, compared with the control groups.

Followed by surface staining with surface markers, the percentages of CD3+ CD8+ CD4-T cell subset and CD3+ CD4+ CD8-T cell subset in spleens of mice from each group were analyzed by flow cytometry. As shown in Figure 2, the percentages of the CD3+ CD8+ CD4-T and CD3+ CD4+ CD8-T lymphocyte subsets in mice immunized with pVAX-ROP5 or pVAX-ROP18 were much higher than those in the blank, PBS, pVAX I control group (*p* < 0.05). Also, the combination of pVAX-ROP5 and pVAX-ROP18 showed a higher percentage of the CD3+ CD8+ CD4-T and CD3+ CD4+ CD8-T lymphocyte subsets than the percentage induced by DNA immunization with pVAX-ROP5 or pVAX-ROP18 alone. Moreover, co-injection of pVAX-IL-33 and pVAX-ROP5 + pVAX-ROP18 boosted this immune response induced by multiple-gene DNA immunization.

### Cytokine production

**Figure 3:**
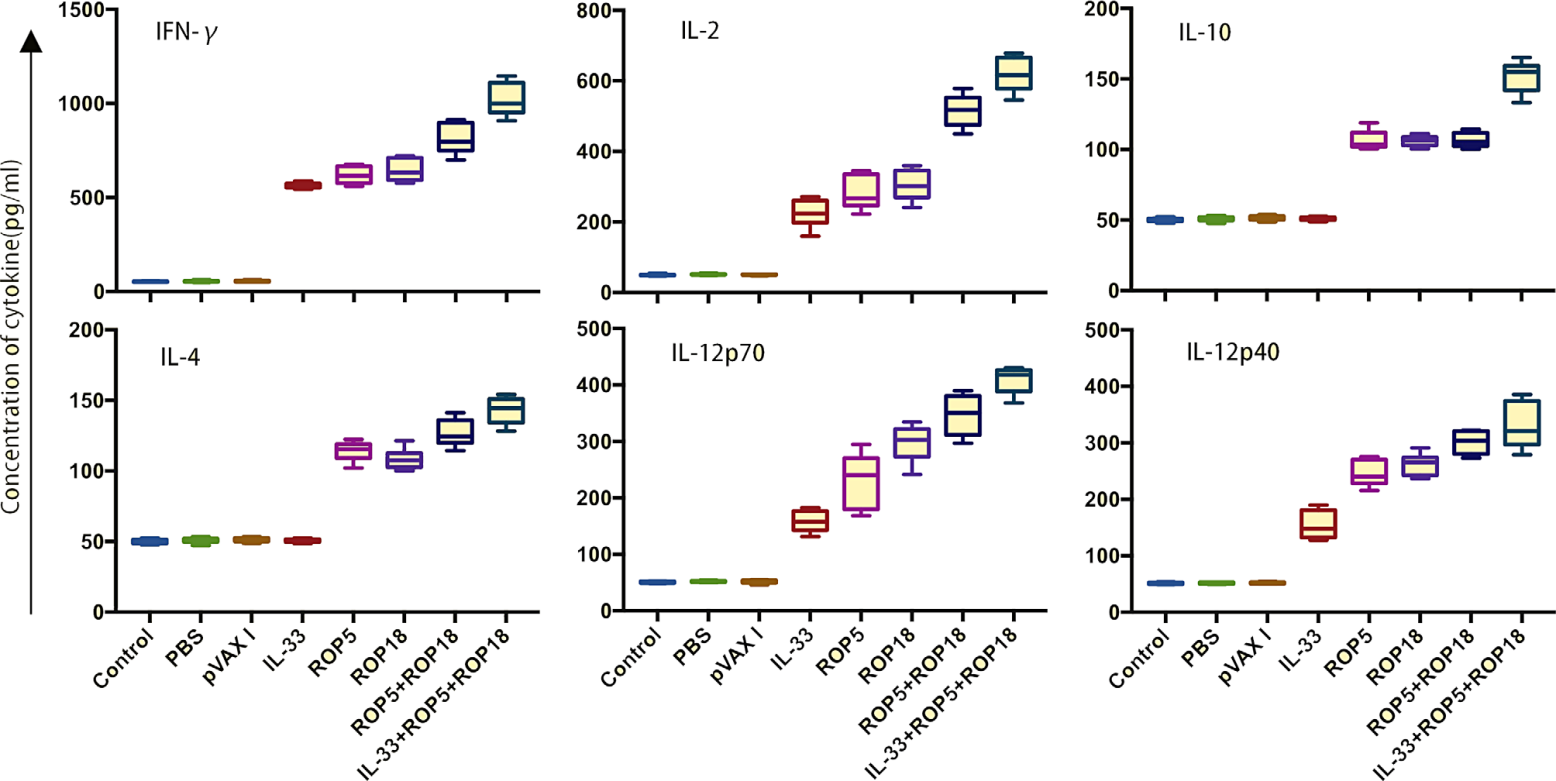
Cytokine production by splenocytes of immunized Kunming mice after stimulation by toxoplasma lysate antigen (TLA). Each bar represents mean pg/mL (±SE, *n* = 3). *p < 0.05.

Supernatants were collected from splenocytes, which were harvested from immunized mice and non-immunized mice two weeks after the last immunization and cultured under TLA stimulation, and were then used to evaluate the amount of cytokines, including IFN-γ, IL-2, IL-12p70, IL-12p40, IL-10, IL-4, and IL-10. Similarly to the results for the flow cytometry analysis and antibody detection, the productions of IFN-γ and IL-2 in all immunized mice were significant higher than in controls. Moreover, smaller amounts of IL-12p70 and IL-12p40 were detected in mice in the experimental groups compared to the control groups (*p* < 0.05). The increased productions of IFN-γ, IL-2, IL-12p70, and IL-12p40 in all immunized mice were similar to the results of the flow cytometry analysis and antibody detection. Also, the levels of IL-4 and IL-10 were increased in supernatants from spleen cells from the pVAX-ROP5, pVAX-ROP18, pVAX-ROP5 + pVAX-ROP18, and pVAX-IL-33 + pVAX-ROP5 + pVAX-ROP18 groups compared to the controls (*p* < 0.05) (Fig. 3). However, the levels of IL-4 and IL-10 cytokines showed no statistically significant differences with those in the three controls (*p* > 0.05).

### Protective efficacy in vaccinated mice

**Figure 4:**
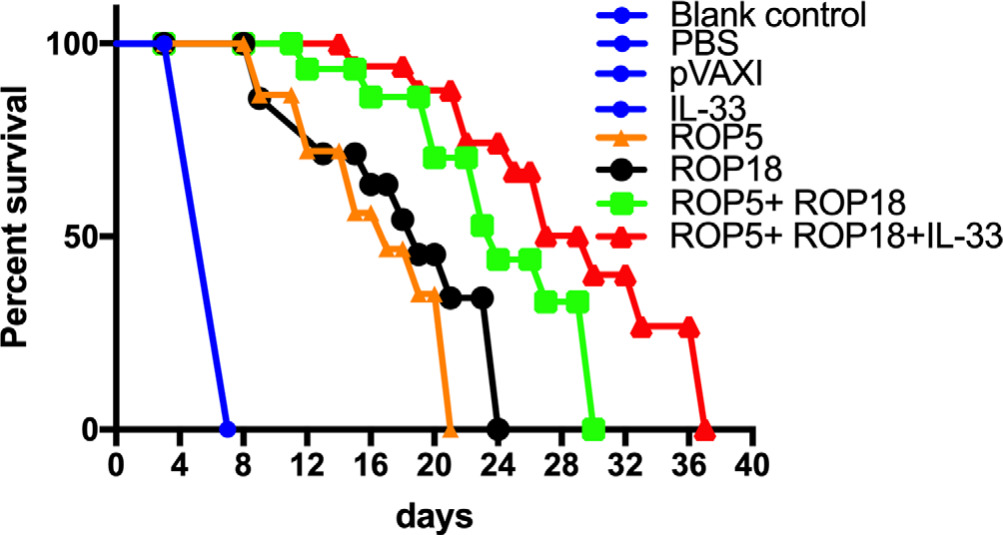
Protection against chronic toxoplasmosis in immunized mice two weeks after the last booster immunization. The bars represent the mean cyst burden per mouse brain after challenge orally with a dose of 10 cysts of the PRU strain. Cyst load was counted from whole brain homogenates of mice four weeks after challenge. Data are means ± SD (representative of three experiments). ***p < 0.001, compared with the control groups.

Mortality was observed daily after challenge with 1 × 10^3^ tachyzoites of the RH strain administered intraperitoneally until all the mice in the experimental and control groups had died. As shown in Figure 4, the immunized mice had a significantly prolonged survival time compared to mice in the PBS, blank and pVAX I control groups. All the mice in the three control groups died within seven days (*p* > 0.05), with 16.4 ± 3.8 days in the pVAX-ROP5 group, 19.6 ± 4.7 days in the pVAX-ROP18 group, 21.1 ± 5.2 days in the pVAX-ROP5 + pVAX-ROP18 group, and 28.7 ± 5.4 days in the pVAX-IL-33 + pVAX-ROP5 + pVAX-ROP18 group (*p* < 0.05) after challenge with 1 × 10^3^ tachyzoites of the RH strain. There was no significant difference among the three control groups (*p* > 0.05).

**Figure 5:**
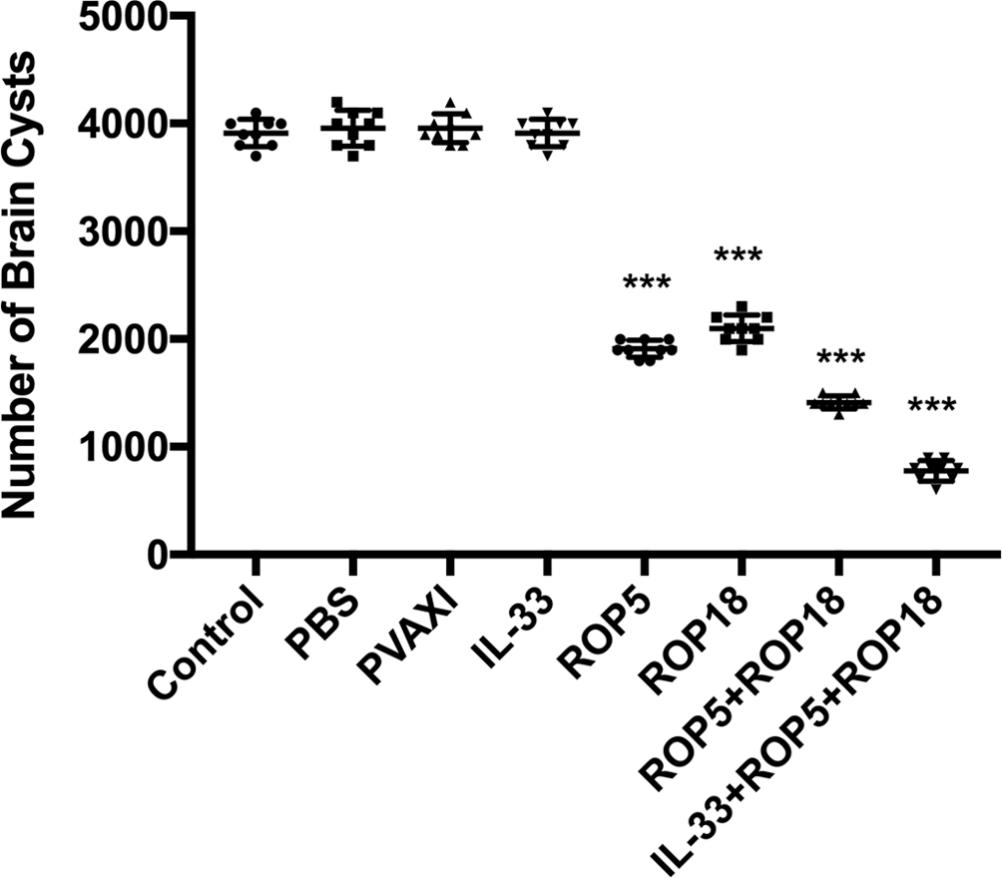
Survival curves of Kunming mice after challenge of *T. gondii* RH strain. The mice (10 per group) in all groups were challenged with 1 × 10^3^ tachyzoites of the virulent *T. gondii* RH strain intraperitoneally. Three control groups (PBS, pVAX I and blank control) had 0% survival at day 7. Immunized groups had prolonged survival time of mice.

To evaluate the protective efficacy against chronic infection with the *T. gondii* PRU strain, tissue cyst loads were detected in brains in experimental mice and controls at four weeks after the third immunization. As shown in Figure 5, there was a significant reduction in the number of tissue cysts in the brains of immunized mice compared to those in the three controls (*p* > 0.05), with the average parasite burden reduced by 51.7%, 46.9%, 64.3%, and 80.3% for the pVAX-ROP5, pVAX-ROP18, pVAX-ROP5 + pVAX-ROP18, and pVAX-IL-33 + pVAX-ROP5 + pVAX-ROP18 groups, respectively.

## Discussion

DNA immunization has been considered an effective approach to induce protection against intracellular parasites, including *T. gondii*, with the ability to induce long-term specific humoral and cell-mediated immunity *in vivo* in animal models [20]. It has been demonstrated previously that a single plasmid encoding ROP5 or ROP18 is a promising candidate used as a the DNA vaccine, resulting in partial protective immunity against *T. gondii* infection [3, 5], but sterilizing immunity has not been achieved. In line with these previous studies, our present study emphasized again that a monovalent vaccine is not sufficient for protection due to the complexity of the *T. gondii* life cycle [39, 40].

A combination of recombinant ROP5 and ROP18 proteins could be used as a valuable component of a subunit vaccine against toxoplasmosis, since a synergistic effect of these two candidates was suggested [10]. However, no synergistic effect after bivalent DNA immunization with pVAX-ROP18 and pVAX-ROP5 was identified. In order to further evaluate the protective efficacy of these two antigens, we mixed a single plasmid pVAX-ROP18 and pVAX-ROP5, and tested whether this could be a potent DNA vaccine against *T. gondii* infection in mice models. In the present study, DNA vaccination with pVAX-ROP5 and/or pVAX-ROP18 induced protective immunity against *T. gondii* infection. Moreover, immunization with pVAX-ROP5 and pVAX-ROP18 increased the tendency toward Th1-type and CD8+ T cell-mediated immune responses induced by injection with pVAX-ROP5 or pVAX-ROP18 alone, resulting in significantly improved survival time and reduced brain cyst burden in comparison with that in DNA immunization with the single-antigen gene plasmid pVAX-ROP18 or pVAX-ROP5 (*p* > 0.05). Therefore, together with previous studies, it has been emphasized again that Th1 immune responses and protective immunity can be enhanced by cocktailed DNA immunization [3, 40]. Also, our results suggest that these two DNA vaccine candidates are attractive cocktailed targets for further validation and we also propose that a suitable adjuvant could boost the immunogenicity of this DNA vaccine cocktail.

As a genetic adjuvant, cytokine genes have been co-administered to enhance the magnitude and nature of the immune responses induced by DNA vaccines [6, 27]. IL-33 is a member of the IL-1 family, historically recognized as a cytokine involved in driving Th2 immune responses, and its actions exceed Th2 immunity, which plays pleiotropic roles in contributed to the development of Th1 and CD8 T cells, particularly targeting intracellular pathogens [2, 19, 29, 30]. Therefore, we examined the potentiality of cytokine gene IL-33 as a potent molecular adjuvant in a vaccine designed to improve protection against *T. gondii* in mice. Our study demonstrated that the addition of IL-33 to the vaccine given to the group with DNA immunization with pVAX-ROP5 and pVAX-ROP18 can improve protective immunity against acute and chronic *T. gondii* infection in mice, followed by boosted humoral immune responses, augmented proliferation of lymphocytes, and up-regulated predominant Th1-biased and CD8+ T cell responses. These findings are consistent with our previous observations, showing that the synergy of IL-21 and IL-15, and the synergy of IL-7 and IL-15 could augment the protective immunity induced by DNA vaccines [4, 17]. Likewise, IL-18 can also act as an immunoadjuvant to improve DNA vaccine-induced protective anti-*T. gondii* Th1-biased responses [36]. Similar outcomes have been shown in which IL-33, as a potent molecular adjuvant, can augment protective antiviral CD8+ T cell responses induced by a vaccine and boost DNA vaccine-induced protective anti-tumor T cell responses [29, 31]. However, unlike the efficacy induced by injection of pVAX-IL-21-IL-15 or pVAX-IL-7-IL-15, our study has found that the administration of pVAX-IL-33 alone could not elicit protective immunity against *T. gondii* infection effectively.

Robust humoral response seems to be required to control *T. gondii* infection during the course of *T. gondii* infection naturally, associated with inhibition of the attachment of the parasite to host cell receptors, as well as opsonizing the parasite for phagocytosis and participating in the activation of the classical complement pathway [24]. In our study, mice in the immunized groups displayed high specific IgG antibody titers than controls; in particular, the combination group with pVAX-ROP5 + pVAX-ROP18 showed higher specific IgG antibody titers than the single DNA immunization group with pVAX-ROP5 or pVAX-ROP18. In addition, DNA immunization induced a significantly higher ratio of IgG2a to IgG1 titers, a characteristic of the Th1-type response in contrast to three control groups. These results are consistent with other studies that used DNA immunization with certain rhoptry proteins, which demonstrated that mice immunized with pVAX-ROP38 or pVAX-ROP5 developed a predominance of specific IgG2a antibodies [3, 35]. However, our observations are contrary to the results for protective immunity induced by recombinant RO5 and ROP18 proteins as components of a subunit vaccine, which elicited a mixed type of immune response with predominant IgG1 synthesis [10]. Furthermore, co-administration of pVAX-IL-33 with DNA vaccines exhibited the highest specific IgG antibodies and ratio of IgG2a–IgG1 compared to DNA vaccines used alone, demonstrating that pVAX-IL-33 was successful in boosting vaccine-elicited specific antibody responses.

Th1-type immune response is thought to play a crucial role in resistance against *T. gondii* infection [9]. IFN-γ, the cytokine of Th1-type lymphocytes, is confirmed to induce inflammatory response and thus to control *T. gondii* load during early stages of infection [9, 13]. Another important Th1-biased cytokine, IL-2 could regulate the proliferation and activities of CTLs, which is important for *T. gondii* resistance [9]. IL-12 is the determinant of Th1 cell immune response, which could promote the production of Th1-biased cytokines such as IFN-γ effectively [13, 15]. IL-23 has the role of promoting the proliferation of T cells and IFN-γ generation, further inducing memory T cell proliferation [21]. In the present study, significantly increased levels of IFN-γ, IL-12p70, IL-23, and IL-2 were detected in all the groups of immunized mice, suggesting that Th1-type mediated immunity was elicited by these DNA candidate injections in mice successfully, which is essential for prolonged survival and reduced brain cysts in the immunized mice. Meanwhile, the combination with pVAX-ROP5 and pVAX-ROP18 boosted this Th1-type-mediated immunity in comparison with a single DNA immunization with pVAX-ROP5 or pVAX-ROP18, emphasizing that a multiple-gene DNA vaccine could elicit greater Th1-type immune responses than a single-gene DNA vaccine [18, 40]. The most significantly improved Th1-biased responses, together with the best Th1-type immune responses and the ratio of IgG2a/IgG were also found in mice immunized with pVAX-ROP5 + pVAX-ROP18 supplemented with pVAX-IL-33, which is similar with the efficacy induced by certain other adjuvant cytokines [4, 17]. Nevertheless, slightly increased levels of IL-4 and IL-10 have been observed in immunized mice, which are associated with Th2-type immune responses, but multiple-gene DNA immunization in synergy with or without pVAX-IL33 has not changed this characteristic induced by single-gene DNA immunization. These results indicate that the specific cellular immune responses induced by these DNA immunizations were primarily skewed to Th1-biased immune responses.

During *T. gondii* invasion, T-cell-mediated immunity is also dominant in the process of host immune response for mediating resistance to *T. gondii* infection [12, 39]. In particular, CD8+ T cells are specialized cytotoxic T lymphocyte cells that mediate lysis of *T. gondii* by the production of IFN-γ or perforin-mediated cytolysis, in synergy with CD4+ T cells [7, 9]. The data in the present study were consistent with some previous reports, underlying the protective effects of these vaccines [18, 35, 38], with the activated proliferative response of lymphocytes and significant increase in both CD8+ and CD4+ T cells in immunized mice. These high levels of CD4+ and CD8+ T cells further emphasized that type-Th1 immune response holds a dominant position in resistance against *T. gondii* infection followed by single-gene DNA immunization, which was also boosted by multiple-gene DNA immunization. Previous studies have shown that the inclusion of IL-33 could increase the frequency of HIV or LCMV-specific CD8+ T cells induced by immunization [30, 31]. In support of these findings, co-injection of pVAX-IL-33 with pVAX-ROP5 + pVAX-ROP18 elicited greater numbers of CD4+ and CD8+ T cells, indicating that this enhanced functionally activated T cells was more beneficial to the development of protective immunity against *T. gondii*.

Previous studies have found that rhoptry proteins ROP5 and ROP18 are major murine virulence factors in globally distributed strains of *T. gondii* [1, 26]. Also, both immunization with ROP5 or ROP18 could induce effective cross-protection between genotypes I and II [16, 32]. Therefore, the primary goal of this DNA vaccine construct is to provide further protective immunity against both acute and chronic toxoplasmosis among different *T. gondii* strains. In this study, we performed the challenge models in mice, with a lethal infection with the wild-type (type I) RH strain, and the low-virulence cyst-forming PRU strain (type II). Our results showed that DNA immunization with pVAX-ROP5 and pVAX-ROP18 induced a significantly longer survival time, and lower brain cyst numbers than a single DNA immunization, suggesting that this combination is an available DNA vaccine cocktail, which is also beneficial for inductive cross-protection between different genotypes of *T. gondii*. These findings support the previous observation, which demonstrated protective efficacy induced by ROP5- or ROP18-based DNA vaccines [3]. However, it is contrary to the protective efficacy induced by recombinant RO5 and ROP18 proteins as components of a subunit vaccine, showing no significant synergetic effect after bivalent immunization with ROP5 + ROP18 [10]. Furthermore, co-delivery IL-33 to this DNA vaccine cocktail enhanced its protective efficacy, demonstrating that IL-33 could be used as a genetic adjuvant to improve protective immunity against *T. gondii* infection.

In conclusion, the present study demonstrates that, for the first time, a potential DNA vaccine cocktail of ROP5 and ROP18, could elicit specific humoral immune responses, and Th1-biased responses against acute or chronic *T. gondii* infection among different strains of *T. gondii* in mice. Co-administration of genetic adjuvant IL-33 enhanced the protective efficacy of this vaccine cocktail by boosting the desired adaptive responses against *T. gondii* challenge, which may facilitate the development of a better vaccine against *T. gondii*, as well as to improve immune responses against other apicomplexan parasites.
